# Smoking Cessation and Alcohol Abstinence: What Do the Data Tell Us?

**Published:** 2006

**Authors:** Suzy Bird Gulliver, Barbara W. Kamholz, Amy W. Helstrom

**Affiliations:** Suzy Bird Gulliver, Ph.D., is outpatient mental health site director at the VA Boston Healthcare System, Brockton Campus, Brockton, Massachusetts, and associate professor in the Departments of Psychiatry and Psychology at Boston University, Boston, Massachusetts. Barbara W. Kamholz, Ph.D., is codirector, VA Boston Healthcare System Mood Disorders Clinic, Jamaica Plain, Massachusetts, and assistant professor of Psychiatry and Psychology at Boston University, Boston, Massachusetts. Amy W. Helstrom, Ph.D., is a clinical psychologist at the VA Boston Healthcare System and Research Associate at Boston University, Boston, Massachusetts

**Keywords:** Alcohol and tobacco, alcohol, tobacco, and other drug (ATOD) use, abuse, and dependence, alcohol and other drug (AOD) craving, AOD use pattern, AOD abstinence, alcohol and tobacco, alcohol abuse, alcoholism, smoking, cigarette smoking, nicotine, treatment program, co-treatment, treatment outcome, AOD abstinence, cue reactivity, alcohol and other drug use disorders (AODD) relapse

## Abstract

Cigarette smoking and nicotine dependence commonly co-occur with alcohol dependence. However, treatment for tobacco dependence is not routinely included in alcohol treatment programs, largely because of concerns that addressing both addictions concurrently would be too difficult for patients and would adversely affect recovery from alcoholism. To the contrary, research shows that smoking cessation does not disrupt alcohol abstinence and may actually enhance the likelihood of longer-term sobriety. Smokers in alcohol treatment or recovery face particular challenges regarding smoking cessation. Researchers and clinicians should take these circumstances into account when determining how best to treat these patients’ tobacco dependence.

Cigarette smoking and alcohol dependence co-occur at high rates. Research indicates that approximately 80 percent of people with alcoholism smoke cigarettes and that most of these smokers are nicotine dependent (Hughes 1996). Conversely, smokers are at two to three times greater risk for alcohol dependence than nonsmokers (Breslau 1995).

## Smoking Cessation and Treatment for Alcoholism

Despite the fact that 60 to 75 percent of patients in alcoholism treatment are tobacco dependent and about 40 to 50 percent are heavy smokers (Hughes 1995), treatment for tobacco dependence is not routinely included in alcohol treatment programs. Smoking cessation treatment (as well as bans on smoking) during the course of treatment for alcohol dependence has been avoided largely out of concern that concurrently addressing both addictions (or restricting smoking during treatment for alcoholism) poses too great a difficulty for the patient and would adversely affect recovery from alcoholism. Such concerns are apparent both in the United States and around the world (e.g., Walsh et al. 2005; Zullino et al. 2003). Myths surrounding concurrent treatment for smoking and alcoholism also include the ideas that smoking is a benign problem relative to alcoholism, that patients with comorbid alcoholism have either no interest or no ability to quit smoking, and that patients will relapse to alcohol if they quit smoking. This article summarizes the scientific findings that address these issues and provides evidence-based responses to common concerns about smoking cessation during alcoholism treatment.

### Myth

Smoking is more benign than alcoholism. The short-term effects of alcoholism may appear more dangerous than those of cigarette smoking. However, mortality statistics suggest that more people with alcoholism die from smoking-related diseases than from alcohol- related diseases (Hurt et al. 1996). In addition, the greater prevalence of smoking in alcohol-dependent versus other populations exacerbates health risks (Bien and Burge 1990; York and Hirsch 1995). Researchers have demonstrated synergistic carcinogenic effects for dual substance dependence. For example, the relative risk of laryngeal cancer has been estimated at 2.1 in heavy smokers, 2.2 in heavy drinkers, and 8.1 in people who are both heavy drinkers and heavy smokers (Hinds et al. 1979).

### Myth

Smokers with comorbid alcoholism have either no interest or no ability to quit smoking. It is interesting to note that although addiction treatment programs routinely address multiple substances of addiction (e.g., alcohol, marijuana, heroin, cocaine), tobacco is frequently the sole excluded substance. The scientific literature also frequently describes treatment of multiple nontobacco substances simultaneously, making it difficult to evaluate the impact of smoking cessation on alcoholism treatment per se (*cf.* Prochaska et al. 2004). Still, evidence contradicts the notion that smokers with comorbid alcoholism are not interested in quitting smoking and that addictions need to be treated one at a time (e.g., Kalman 1998). Up to 80 percent of people in addiction treatment are interested in quitting smoking (*cf.* Prochaska et al. 2004). Consistent with this, Flach and Diener (2004) found that among dual users, approximately 75 percent wanted to quit both smoking and alcohol use (though the desire to quit alcohol use was rated as higher). Furthermore, many people entering treatment for alcoholism are willing to quit smoking (e.g., Saxon et al. 1997). In fact, one study found that 75 percent of substance-dependent inpatients accepted concurrent tobacco treatment (Seidner et al. 1996).

Inclusion of smoking as a target for intervention does not appear to reduce patients’ commitment to broader addiction treatment. For example, incorporating smoking cessation treatment into inpatient addiction treatment centers has not substantially reduced longterm treatment completion (e.g., a minimal drop from 75 to 70 percent at one site) (Sharp et al. 2003). In addition, Monti and colleagues (1995) found that smoking rates actually decrease and the motivation to quit smoking increases following successful alcohol treatment.

Evidence suggests that a history of alcohol use difficulties may not impede a specific smoking cessation attempt, though it does seem to reduce the likelihood of quitting smoking during one’s lifetime (Hughes and Kalman 2005). Research has yet to determine the extent to which smokers with current alcohol use difficulties are able to quit smoking. Though early research has suggested that quitting smoking would be more difficult for these patients (e.g., Hughes 1996), the answer is now less clear. The only two studies evaluating this issue separate from other substances of abuse and co-occurring psychiatric disorders yielded mixed findings and did not include more severe alcohol-dependent individuals (*cf.* Hughes and Kalman 2005). However, studies based on smokers in substance abuse treatment, and those in early recovery, suggest that cigarette abstinence is possible, though challenging (Martin et al. 1997; Prochaska et al. 2004).

**Myths and Data Related to Smoking Cessation and Alcohol Abstinence**

**Myth: Smoking is more benign than alcoholism.**

- More people with alcoholism die from smoking-related diseases than from alcohol-related illness (Hurt et al. 1996).
- Comorbid smoking and alcoholism result in synergistic exacerbation of health risks (Bien and Burge 1990; York and Hirsch 1995; Hinds et al. 1979).

**Myth: Smokers with comorbid alcoholism have either no interest or no ability to quit smoking.**

- The majority (up to 80 percent) of individuals in addiction treatment are interested in quitting smoking (*cf.* Prochaska et al. 2004).
- Inclusion of smoking cessation treatment into other addiction programs does not negatively affect rates of treatment completion or motivation for abstinence (Sharp et al. 2003; Monti et al. 1995).
- Alcoholism does not seem to impede specific attempts at quitting smoking (Hughes and Kalman 2005).
- Alcoholism may make lifetime cigarette abstinence more challenging, but it remains possible (Martin et al. 1997; Prochaska et al. 2004).

**Myth: Smoking cessation will impede successful alcohol use outcomes.**

- The majority of research indicates that smoking cessation is unlikely to compromise alcohol use outcomes (*cf*. Fogg and Borody 2001).
- Participation in smoking cessation efforts while engaged in other substance abuse treatment has been associated with a 25 percent greater likelihood of long-term abstinence from alcohol and other drugs (Prochaska et al. 2004).
- Data indirectly suggest that continued smoking increases the risk of alcohol relapse among alcohol-dependent smokers (Taylor et al. 2000).

### Myth

Smoking cessation will impede successful alcohol use outcomes. Perhaps most important is the concern among treatment providers (and patients) that patients must choose between abstinence from cigarettes and abstinence from alcohol. In contrast to this concern, research suggests that treating tobacco dependence within broader addiction programs does not adversely influence recovery from alcoholism (or illicit substances). Although not universal (e.g., Joseph et al. 2004), the majority of findings indicate that smoking cessation efforts and smoking abstinence are unlikely to negatively influence alcohol use outcomes (*cf.* Fogg and Borody 2001). In a recent meta-analysis, Prochaska and colleagues (2004) evaluated the outcomes of smoking cessation interventions in 19 randomized controlled trials with people in addiction treatment or recovery. At the end of treatment, no differences in substance use outcomes were found between patients who engaged in smoking cessation treatment and those who did not. Looking at long-term abstinence from substances, an even more important finding emerged. That is, at long-term follow-up, participation in a smoking cessation intervention provided during substance abuse treatment was associated with a 25 percent greater likelihood of long-term abstinence from alcohol and other drugs. Consistent with these findings, data suggest that 1 year after treatment, smokers who participated in a substance abuse treatment program and initiated smoking cessation on their own were less likely to be diagnosed as alcohol dependent and had more days abstinent from alcohol and other substances than those who started or continued smoking during the follow-up period (Kohn et al. 2003). Thus, empirical evidence suggests that smoking cessation efforts may result in improved alcohol-related outcomes (even if those efforts do not yield substantial smoking abstinence).

The mechanisms of action responsible for the potential benefits of smoking cessation interventions provided during alcoholism treatment remain largely unexplored. However, possible explanatory factors may include greater clinical contact time, reduced exposure to substance use cues, relapse prevention and/or coping skills practice, increased mastery or self-efficacy, and broader healthy lifestyle choices (Prochaska et al. 2004). Self-initiated efforts to reduce smoking also may reflect increased patient motivation or lower levels of nicotine dependence (Karam-Hage et al. 2005).

Alcohol-dependent patients who quit smoking while in recovery from alcohol problems also do so without negative consequences to their alcohol or drug abstinence (Bien and Burge 1990; Bobo 1989; Hurt et al. 1993; Irving et al. 1994; Joseph et al. 2003; Sobell et al. 1990; Sullivan and Covey 2002). Data suggest that among alcohol-dependent smokers in early recovery, nicotine deprivation is not associated with an increased urge to drink. In addition, among people with significant alcohol abstinence, evidence suggests that smoking cessation does not increase the likelihood of relapse to alcohol use or increase alcohol-related cravings (Hughes et al. 2003). Data from Project MATCH, the largest alcoholism clinical trial published to date, indicates that alcohol-dependent smokers can quit smoking cigarettes without putting their sobriety at risk. In fact, those who quit smoking during their participation in Project MATCH drank less than those who did not quit smoking and significantly reduced their alcohol intake for the 6 months after quitting smoking (Friend and Pagano 2005). Similarly, Karam-Hage and colleagues (2005) studied smokers in alcohol treatment and found that participants who quit smoking on their own were more likely to report alcohol abstinence at 1- and 6-months’ followup than participants who did not quit smoking (though this may be a function of lower levels of nicotine dependence).

Not only does the preponderance of evidence suggest that smoking cessation does not compromise alcohol abstinence, but multiple studies indirectly suggest that continued smoking may place alcohol-dependent smokers at risk for alcohol relapse (Taylor et al. 2000). These data are consistent with laboratory studies on cross-cue reactivity, which suggest that nicotine dependence and alcoholism may interact to increase drinking risk. For example, alcohol cues, such as the sight or smell of an alcoholic beverage, can increase smoking urges among smokers with alcohol use disorders (e.g., Cooney et al. 2003; Drobes 2002; Gulliver et al. 1995; Rohsenow et al. 1997), and the degree of nicotine dependence among alcoholic smokers is positively related to alcohol cue reactivity (Abrams and Biener 1992). In addition, a study of hazardous drinkers (i.e., those scoring 8 or above on the Alcohol Use Disorders Identification Test [Babor et al. 1992]) found that 6 hours of nicotine deprivation was associated with increased alcohol cravings during exposure to smoking cues (e.g., cigarette lighter, ashtray, pack of favorite cigarettes) as well as increased alcohol consumption during a taste test procedure (Palfai et al. 2000). Alcohol cravings also were increased during neutral cue exposure, suggesting that stopping one drug of abuse and not another may result in cross-cue reactivity that places a person in recovery at increased risk for relapse (Bobo et al. 1998; Toneatto el al. 1995).

## Challenges in Treating Co-Occurring Smoking and Alcoholism

Unfortunately, even with today’s best interventions for tobacco cessation, smokers in alcohol treatment or recovery face particular challenges to their cessation efforts. On average, compared with smokers who do not abuse substances, alcoholic smokers are more addicted to nicotine, smoke higher nicotine cigarettes, smoke more per day, and score higher on nicotine dependence measures and on carbon monoxide assessment (Burling and Burling 2003; York and Hirsch 1995). Many smokers with alcoholism report that they use smoking to cope with their urges to use alcohol or other drugs (Rohsenow et al. 2005), so alcohol-dependent smokers may have stronger views about the benefits of continued tobacco use than do other smokers. In addition, nicotine positively influences information processing among alcoholics (i.e., nicotine use increases the speed and accuracy of information processing) (Ceballos et al. 2006), which may decease motivation to change. Thus, researchers and clinicians must take into account the characteristics of tobacco dependence in alcohol-dependent populations when determining how best to treat these patients’ tobacco dependence.

## Summary

Despite concerns to the contrary, the majority of empirical evidence indicates that smoking cessation (whether through formal treatment or self-initiated change) does not pose a risk to successful alcoholism treatment. Not only does smoking cessation not disrupt alcohol abstinence, it actually may enhance the likelihood of longer-term sobriety. Although research has yet to determine the extent to which smoking cessation is impeded by active alcohol use difficulties, the presence of these difficulties does not prohibit achievement of tobacco abstinence. Given the substantial negative health consequences of co-occurring cigarette smoking and alcoholism, smoking cessation efforts in the context of treatment for alcoholism are likely to yield important benefits to patients physically, emotionally, socially, and economically.
